# Characteristics of non-stenotic carotid plaque in embolic stroke of undetermined source compared with cardiogenic embolism: a retrospective cross-sectional observational study

**DOI:** 10.1186/s12883-022-02846-4

**Published:** 2022-08-25

**Authors:** Lihao Zhang, Yikun Guo, Wang Zhou, Shumin Zhu, Zhuoyou Chen, Guanzhong Dong, Yin Cao

**Affiliations:** 1 Department of Neurology, The Affiliated Changzhou No.2 People’s Hospital of Nanjing Medical University, 68# Middle Gehu Road, Changzhou, 213164 Jiangsu Province People’s Republic of China; 2 Department of Psychology, The Affiliated Changzhou No.2 People’s Hospital of Nanjing Medical University, 68# Middle Gehu Road, Changzhou, 213164 Jiangsu Province People’s Republic of China

**Keywords:** Non-stenotic carotid plaque, Embolic stroke of undetermined source, Ultrasound, Cardiogenic embolism

## Abstract

**Background:**

Non-stenotic carotid plaque is considered an important etiology of embolic stroke of undetermined source (ESUS). However, only a few previous studies included a negative control group, and the characteristics of non-stenotic carotid plaque in ESUS have yet to be investigated. The objective of this study is to explore the clinical characteristics of ESUS and the correlation between non-stenotic carotid plaque and ESUS.

**Methods:**

This is a single-center, retrospective cross-sectional observational study conducted to compare differences in clinical information among ESUS, CE, and large-artery atherosclerosis (LAA), as well as the prevalence of non-stenotic carotid plaque and non-stenotic carotid plaque with low echo between patients with ESUS and CE in Changzhou No.2 People’s Hospital from January 2020 to January 2022. Ultrasound was used to evaluate the characteristics of non-stenotic carotid plaque and vulnerable carotid plaque was defined as plaque with low echo. The binary logistic regression model was used to analyze the relationship between the characteristics of non-stenotic carotid plaque and ESUS. The receiver-operating characteristic curve was used to evaluate the diagnostic efficiency of the characteristics of non-stenotic carotid plaque for ESUS.

**Results:**

We had a final studying population of 280 patients including 81 with ESUS, 37 with CE, and 162 with LAA. There were no differences in clinical features between ESUS and LAA, but in the comparison of CE and ESUS, there were differences in age, smoking, hypertension, levels of triglyceride, total cholesterol, and low density lipoprotein cholesterol. In ESUS, the prevalence of non-stenotic carotid plaque was more common on the ipsilateral side of stroke than in CE [55 (67.90%) vs. 18 (48.65%), *p* = 0.046], so was the prevalence of non-stenotic carotid plaque with low echo [38 (46.91%) vs. 5 (13.51%), *p* < 0.001]. Logistic regression analysis showed that the prevalence of non-stenotic carotid plaque (OR: 4.19; 95% CI: 1.45–12.11; *p* = 0.008) and the prevalence of non-stenotic carotid plaque with low echo (OR: 5.12; 95% CI: 1.55–16.93; *p* = 0.007) were, respectively, the independent predictors of ESUS. The results receiver-operating characteristic (ROC) curve showed that the combination of age, hypertension, and ipsilateral non-stenotic carotid plaque with low echo had the best diagnostic efficiency for ESUS (0.811; 95%CI: 0.727–0.896; *p* < 0.001).

**Conclusion:**

Our results suggest that ipsilateral vulnerable non-stenotic carotid plaque is associated with ESUS in anterior circulation infarction.

## Introduction

Embolic stroke of undetermined source (ESUS) refers to the non-lacunar infarct pattern on computed tomography (CT) or magnetic resonance imaging (MRI) without obvious luminal stenosis (vessel stenosis ≥50%) in the arteries supplying the infarct region and significant cardioembolic source. Reportedly, it involves approximately 17% of all ischemic stroke and could be attributed to a variety of causes [1].

Studies have shown that an appreciable incidence of atrial fibrillation (AF) was detected during the follow-up, and the occult AF is considered to be the most common etiology of ESUS [2]. However, the results of studies focusing on anticoagulant therapy in ESUS patients were neutral [3, 4]. Recently, the prevalence of non-stenotic carotid plaques in ESUS patients was found higher on the ipsilateral side than on the contralateral side, suggesting that the non-stenotic carotid plaques might be another cause of ESUS [3, 5].

Ultrasonography is one of the most commonly used techniques in evaluating carotid plaque. Former studies have shown that plaque with low echo is often correlated to the presence of lipid-rich necrotic cores. The plaque with low echo was found in approximately 50% of symptomatic plaques but only 5% of asymptomatic plaques. Additionally, regardless of the degree of stenosis, patients with plaques with low echo had a higher risk of stroke than patients with high-grade stenosis. As a result, the plaque with low echo in ultrasonography indicates plaque vulnerability [6].

The clinical information of ESUS, cardiogenic embolism (CE), and large-artery atherosclerosis (LAA) were compared in this study to see if the risk factors in ESUS are more similar to LAA rather than to CE. This study also used carotid ultrasonography to assess the characteristics of non-stenotic carotid plaque in patients with ESUS and cardiogenic embolism (CE) to confirm the association between the ipsilateral non-stenotic carotid plaques and ESUS and the association is not present in CE.

## Materials and methods

### Study population

We retrieved data from the stroke center database of Changzhou No.2 People’s Hospital, Jiangsu Province, China. This study was approved by the Clinical Research Ethics Committee of the Affiliated Changzhou No.2 People’s Hospital of Nanjing Medical University (2021KY312–01). A written informed consent was obtained from the patients or their legally authorized representatives. All patient information was de-identified. This study was conducted in accordance with the Declaration of Helsinki.

Consecutive acute ischemic stroke patients with the diagnosis of LAA, CE, and ESUS from January 2020 to January 2022 were retrospectively enrolled. Patients were included if they met the following criteria: (1) age ≥ 18 years old, (2) unilateral anterior circulation infarction, (3) Necessary assessments were performed for the diagnosis of stroke subtypes such as hematologic screening, brain CT or MRI, 12-lead ECG, ≥24 h dynamic electrocardiogram, precordial echocardiography, extra- and intra-cranial angiography and carotid ultrasonography. Patients were excluded if they (1) undertook thrombectomy, balloon dilatation or stent, (2) had posterior or bilateral infarcts on DWI, (3) were diagnosed as other causes of stroke (such as arteritis, dissection, and vasospasm) or (4) combined with intracranial hemorrhagic disease, central nervous system infections, or tumors.

ESUS was identified as: (1) stroke detected by CT or MRI that is not lacunar, (2) absence of extracranial or intracranial atherosclerosis causing ≥50% luminal stenosis in arteries supplying the area of ischemia, (3) no major-risk cardioembolic source of embolism, (4) no other identified specific cause of stroke [7]. Diagnosis for LAA, CE, and other stroke subtypes were made according to the TOAST classification [8]. Two senior neurologists were responsible for the patients’ recruitment, and if there was disagreement, an agreement was reached through discussion.

### Assessment of carotid plaques

Characteristics of common and internal carotid plaques in patients with ESUS and CE were assessed using a Philips IE33 duplex ultrasonographic device (USA) and plaque echogenicity was classified into high echo (calcified lesions), mixed echo (combined with calcified and hypoechoic lesions) and low echo (high lipid or hemorrhage lesion). Operators all followed a standard operating method and process. Carotid plaque was defined as minimal intima-media thickness ≥ 1.2 mm. Vulnerable carotid plaque was defined as plaque with low echo [9].

### Statistical analysis

Statistical analyses were conducted by SPSS 23.0. Continuous variables in normal distribution were given as mean and SD otherwise as median and interquartile. Pairwise comparisons were performed by the Student’s t test or Mann–Whitney test for continuous variables and Chi square test for categorical variables. Association between ipsilateral non-stenotic carotid plaque and ESUS was analyzed using binary logistic regression. The receiver-operating characteristic (ROC) curve was used to evaluate the diagnostic efficiency of the characteristics of non-stenotic carotid plaque combined with other risk factors for ESUS. *P* < 0.05 was taken to be statistically significant. Figures were created using GraphPad Prism 9.

## Results

### Baseline information

**Fig. 1:**
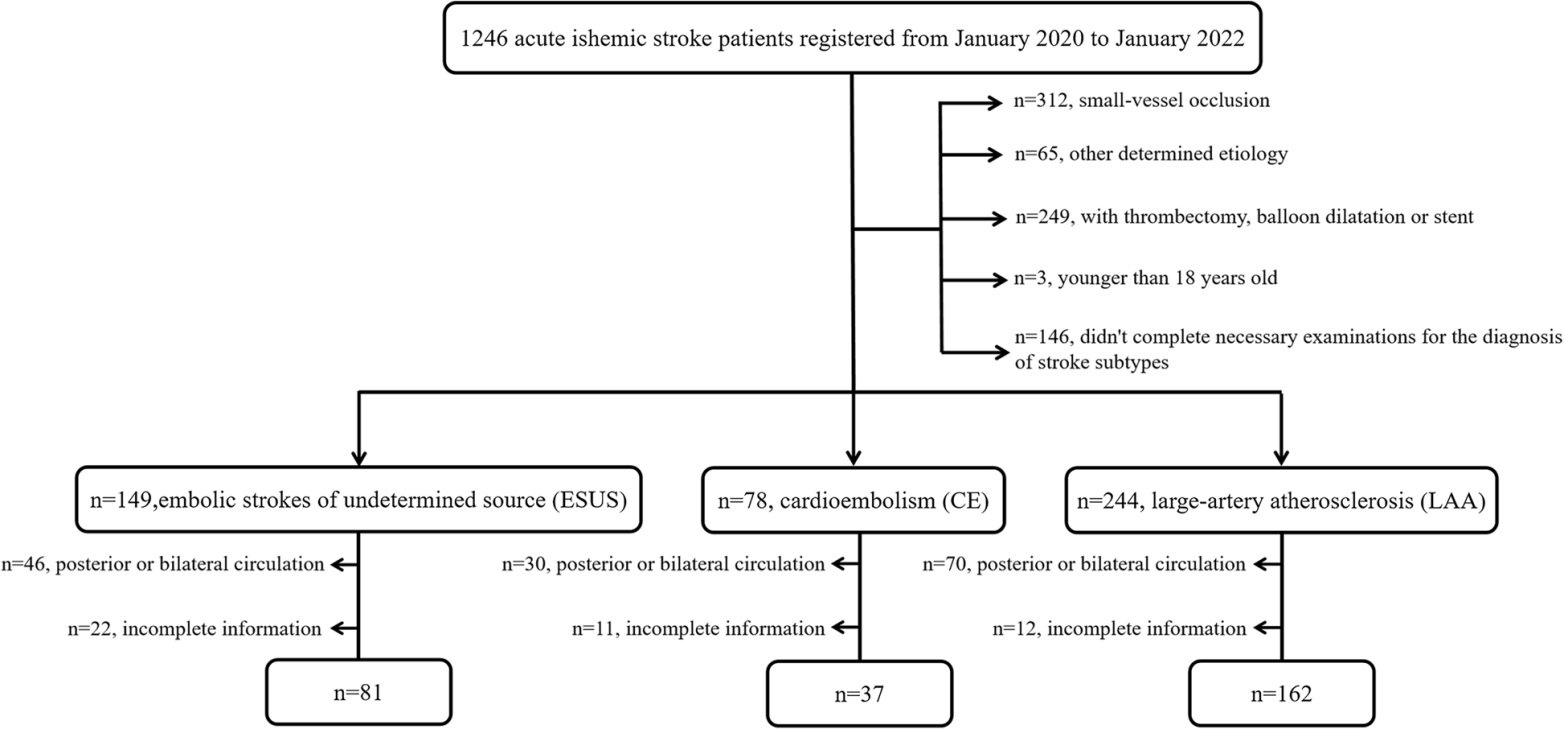
The flow chart of the study recruitment

A total of 471 patients were enrolled, including 149 ESUS patients, 78 CE patients, and 244 LAA patients. For ESUS group, 46 patients who had posterior or bilateral infarcts and 22 patients who had incomplete information were excluded. For CE group, 30 patients with posterior or bilateral infarcts and 11 patients without complete information were excluded. For LAA group, 70 patients with posterior or bilateral infarcts and 12 patients with incomplete information were excluded. Finally, 280 patients were included for analysis, of whom 81 had ESUS, 37 had CE, and 162 had LAA (Fig. 1).

### Differences of risk factors between groups

In comparison to CE patients, ESUS patients were younger (*p* = 0.001), more likely to be a smoker (40.74% vs. 18.92%, *p* = 0.02), and had a higher prevalence of hypertension (82.72% vs. 59.46%, *p* = 0.006). Also, they had a higher level of triglyceride (TG) (*p* < 0.001), total cholesterol (TCH) (*p* = 0.027), and low density lipoprotein cholesterol (LDL-C) (*p* = 0.044). There was no difference in neurological deficit measured using the National Institutes of Health Stroke Scale (NIHSS) between ESUS and CE. During the screening, we found 13 (16.05%) patients in ESUS and 5 (13.51%) patients in CE with non-stenotic intracranial plaque ipsilateral to the stroke site, with no difference (*p* = 0.722).

When comparing ESUS patients with LAA patients, there were no differences in clinical features between the two groups. Since the etiology of LAA is stenosis or occlusion of large arteries, we did not count the plaque in LAA patients. Detailed information can be seen in Table 1.

**Table 1:** Basic information of patients with anterior circulation stroke in ESUS, CE, and LAA

**Characteristics**	n**CE (*n* = 37)**	**ESUS (*n* = 81)**	***P*-value**	**LAA (*n* = 162)**	**ESUS (*n* = 81)**	***P*-value**
Gender male, n (%)	22 (59.46)	51 (62.96)	0.716^a^	101 (62.35)	51 (62.96)	0.925^a^
Age, years, median (IQR)	74.0 (64.0–82.5)	65.0 (56.0–72.5)	0.001^b^	67.0 (57.8–74.0)	65.0 (56.0–72.5)	0.302^b^
Smoking, n (%)	7 (18.92)	33 (40.74)	0.020^a^	56 (34.57)	33 (40.74)	0.346^a^
Diabetes Mellitus, n (%)	10 (27.03)	31 (38.27)	0.234^a^	72 (44.44)	31 (38.27)	0.359^a^
Hypertension, n (%)	22 (59.46)	67 (82.72)	0.006^a^	141 (87.04)	67 (82.72)	0.366^a^
Homocysteine, μmol/L, mean ± SD	10.97 ± 3.48	11.74 ± 7.09	0.534^c^	11.25 ± 6.32	11.74 ± 7.09	0.588^c^
Triglyceride, mmol/L, mean ± SD	1.03 ± 0.55	1.56 ± 0.90	< 0.001^c^	1.56 ± 1.01	1.56 ± 0.90	0.996^c^
Lipoprotein (a), g/L, mean ± SD	0.25 ± 0.19	0.27 ± 0.24	0.661^c^	0.32 ± 0.28	0.27 ± 0.24	0.165^c^
Total cholesterol, mmol/L, mean ± SD	3.81 ± 0.68	4.21 ± 0.99	0.027^c^	4.06 ± 0.93	4.21 ± 0.99	0.262^c^
High density lipoprotein cholesterol, mmol/L, mean ± SD	1.15 ± 0.31	1.07 ± 0.22	0.177^c^	1.05 ± 0.26	1.07 ± 0.22	0.597^c^
Low density lipoprotein cholesterol, mmol/L, mean ± SD	2.24 ± 0.59	2.55 ± 0.81	0.044^c^	2.46 ± 0.76	2.55 ± 0.81	0.425^c^
NIHSS score, median (IQR)	4.0 (3.0–5.0)	3.0 (1.0–5.0)	0.107^b^	3.5 (2.0–6.0)	3.0 (1.0–5.0)	0.095^b^
With intracranial plaque ipsilateral to the stroke site, n (%)	5 (13.51)	13 (16.05)	0.722^a^	_	13 (16.05)	_

^a^Pearson’s χ^2^ test

^b^Mann–Whitney U-test

^c^Student’s t-test

*Abbreviations*: *ESUS* Embolic stroke of undetermined source, *LAA* Large-artery atherosclerosis, *CE* Cardiogenic embolism, *NIHSS* National Institutes of Health Stroke Scale, *IQR* Interquartile range, *SD* Standard deviation

### Comparisons of ultrasonic findings between ESUS and CE patients

Compared with CE patients, ESUS patients had a higher incidence of non-stenotic carotid plaques on the ipsilateral side of stroke than that on the contralateral side (67.90% vs. 48.65%, *p* = 0.046). Furthermore, they had a higher incidence of vulnerable ipsilateral non-stenotic carotid plaques than CE patients (46.91% vs. 13.51%, *p* < 0.001). Detailed information can be seen in Table 2.

**Table 2:** Characteristics of non-stenotic plaques in patients with anterior circulation stroke in ESUS and CE

**Characteristics**	**ESUS (*n* = 81)**	**CE (*n* = 37)**	***P*-value**
non-stenotic carotid plaque ipsilateral to the stroke site	55 (67.90)	18 (48.65)	0.046^a^
non-stenotic carotid plaque with low echo ipsilateral to the stroke site	38 (46.91)	5 (13.51)	< 0.001^a^

^a^Pearson’s χ^2^ test

*Abbreviations*: *ESUS* Embolic stroke of undetermined source, *CE* Cardiogenic embolism

### Association between characteristics of carotid plaques and ESUS

**Fig. 2:**
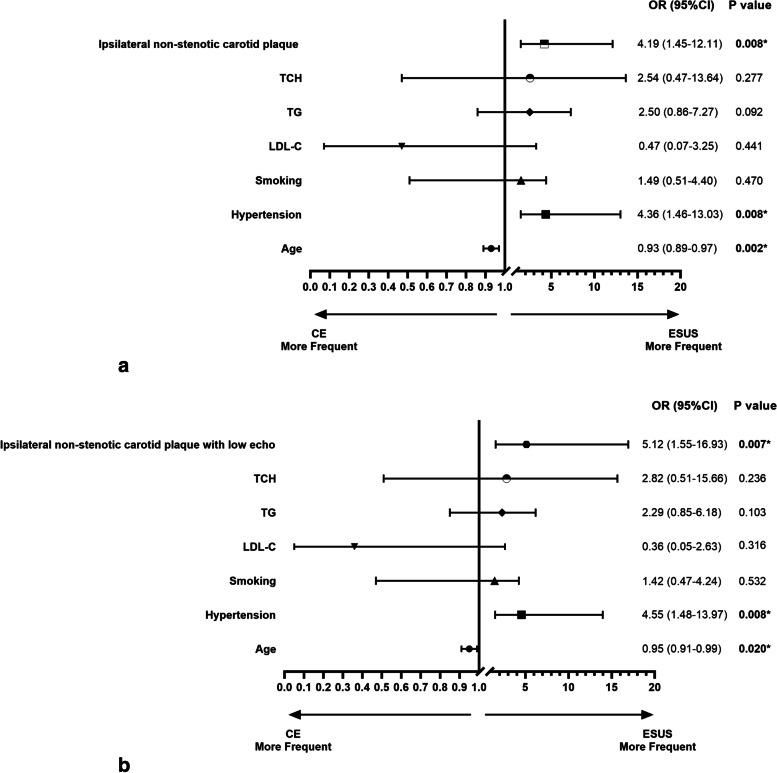
The results of logistic regression analysis. (A) The logistic regression analysis of the model of ipsilateral non-stenotic carotid plaque. (B) The logistic regression analysis of the model of ipsilateral non-stenotic carotid plaque with low echo. Abbreviations: LDL-C, low density lipoprotein cholesterol; TG, triglyceride; TCH, total cholesterol; ESUS, embolic stroke of undetermined source; CE, cardiogenic embolism; OR, odds ratio; CI, confidence interval

After adjustment for variables with *p* < 0.05 in the comparison of ESUS with CE, including age, hypertension, smoking, and hyperlipidemia, the presence of ipsilateral non-stenotic carotid plaque (OR: 4.19; 95% CI: 1.45–12.11; *p* = 0.008), as well as the presence of ipsilateral non-stenotic carotid plaque with low echo (OR: 5.12; 95% CI: 1.55–16.93; *p* = 0.007), remained a predictor for ESUS in binary logistic regression analysis (Fig. 2).

**Fig. 3:**
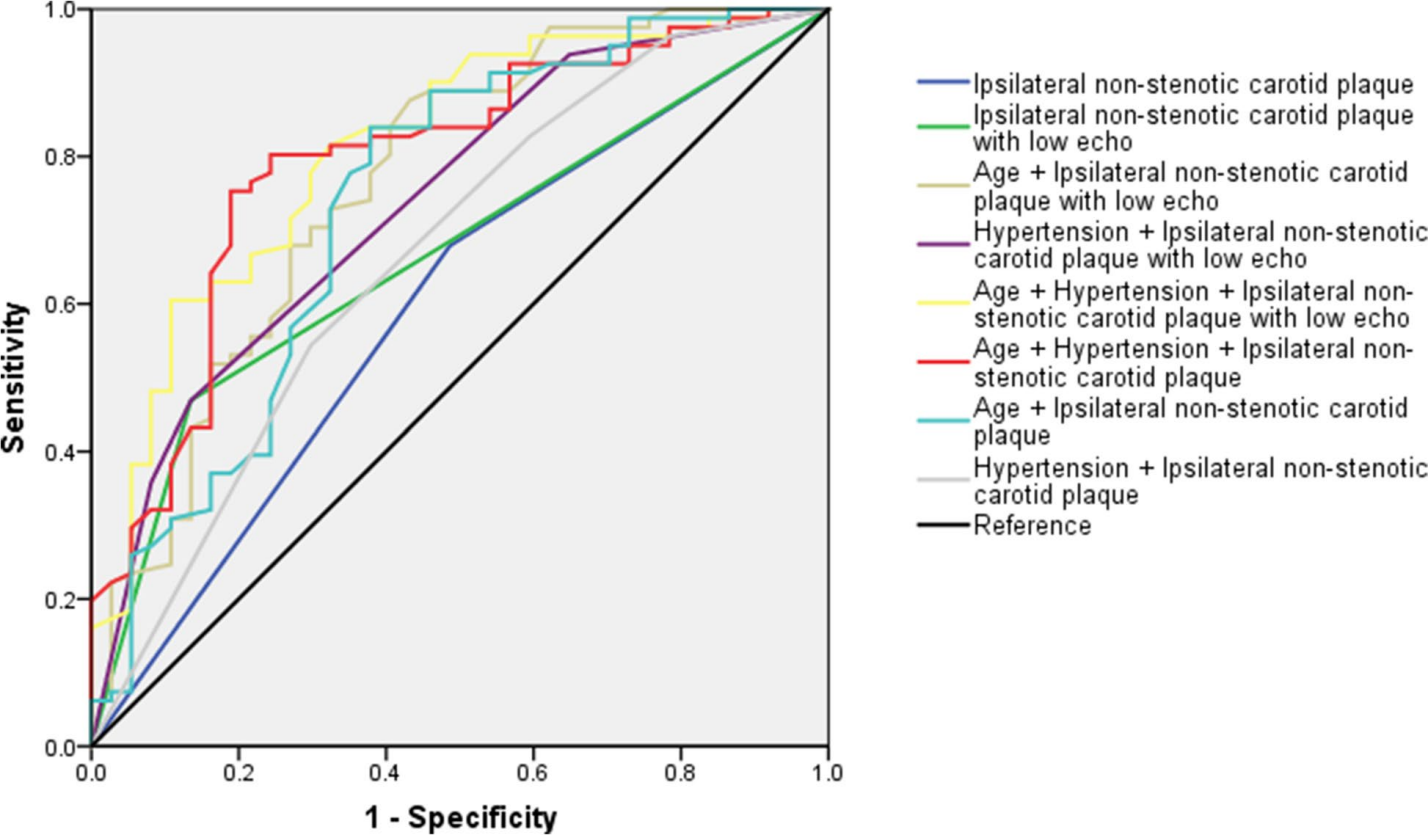
The results of receiver-operating curves which shows the characteristics of non-stenotic carotid plaque in prediction of embolic stroke of undetermined source

In the ROC analysis, variables with *p* < 0.05 in the regression analysis were included. Figure 3 showed the results of ROC analysis: combination of age, hypertension, and ipsilateral non-stenotic carotid plaque with low echo (0.811; 95%CI: 0.727–0.896; *p* < 0.001); combination of age, hypertension, and ipsilateral non-stenotic carotid plaque (0.793; 95%CI: 0.705–0.882; *p* < 0.001); combination of age and ipsilateral non-stenotic carotid plaque with low echo (0.767; 95%CI: 0.670–0.864; *p* < 0.001); combination of hypertension and ipsilateral non-stenotic carotid plaque with low echo (0.739; 95%CI: 0.643–0.835; *p* < 0.001); combination of age and ipsilateral non-stenotic carotid plaque (0.737; 95%CI: 0.633–0.842; *p* < 0.001); ipsilateral non-stenotic carotid plaque with low echo (0.667; 95%CI: 0.567–0.767; *p* = 0.004); combination of hypertension and ipsilateral non-stenotic carotid plaque (0.666; 95%CI: 0.557–0.775; *p* = 0.004); ipsilateral non-stenotic carotid plaque (0.596; 95%CI: 0.484–0.708; *p* = 0.094), suggesting the presence of ipsilateral vulnerable non-stenotic carotid plaque tended to have good diagnostic efficiency for ESUS.

## Discussion

This study explored the clinical characteristics of ESUS patients and used ultrasound to examine the features of non-stenotic carotid plaque in ESUS and CE patients. The results showed that the risk factors for ESUS were more similar to those for LAA than for CE and the non-stenotic carotid plaque was more prevalent and vulnerable on the ipsilateral side of stroke in ESUS than in CE. Moreover, the presence of ipsilateral non-stenotic carotid plaque and ipsilateral vulnerable non-stenotic carotid plaque were, respectively, independent risk factors for ESUS.

Increasing attention has been paid to the atherosclerotic factors in ESUS. Previous studies have found that ipsilateral non-stenotic carotid plaque is more common in ESUS than in other stroke subtypes. Further research discovered that in ESUS patients, some non-stenotic carotid plaques with vulnerable characteristics, such as large size, intraplaque hemorrhage, and low echo, were more likely to appear on the ipsilateral side of the lesion [10–15]. Although there is no negative control in these researches, these results imply that ipsilateral carotid plaques may be associated with ESUS. In line with these findings, we found that ESUS patients had a higher prevalence of ipsilateral non-stenotic carotid plaque and ipsilateral vulnerable non-stenotic carotid plaque than CE patients. Further, we discovered that the combination of age, hypertension, and ipsilateral vulnerable non-stenotic carotid plaque has the best diagnostic efficiency for ESUS. Our findings, together with the results of the studies mentioned above, may suggest that ipsilateral non-stenotic carotid plaque is a potential cause of ESUS. In addition, recent research suggested that complicated aortic arch, as well as intracranial atherosclerotic plaque, were more frequently detected on the ipsilateral side of ESUS [16, 17]. Lately, the term “supracardiac atherosclerosis” was brought up to summarize all the possible sources of arterial embolism on ESUS [18]. All of the findings support the notion that arterial-to-arterial embolization, rather than hemodynamic disruption, is an important mechanism of ESUS produced by non-stenotic carotid plaque.

Clinical investigations have shown that ESUS patients with non-stenotic carotid plaque have a low risk of developing AF or patent foramen ovale (PFO). A pooled analysis of three prospective stroke registries showed that AF was detected in 14.4% of ESUS patients during the follow-up, including 8.5% of patients with the non-stenotic carotid plaque and 19% of patients without [19]. Another larger clinical study demonstrated that only 3.4% of ESUS patients were diagnosed clinically with AF during the trial follow-up period [20]. Similarly, studies have shown that 5–9% of ESUS patients with non-stenotic carotid plaque have PFO [21]. Our study was a cross-sectional study without long-term follow-up and we did not perform transesophageal ultrasonography in the patient screening, the impact of non-stenotic carotid plaque on ESUS might be overestimated. Nevertheless, given the low probability of AF and PFO in ESUS patients, our results still have great clinical significance.

Ultrasound was used in our study to identify risk factors for ESUS. Because of its noninvasive character, convenience, and low cost, ultrasound has been widely used in the risk stratification of stroke. In a study that used multimodal ultrasound to investigate plaque risk stratification in patients with asymptomatic carotid stenosis, plaque surface morphology, intraplaque neovascularization grades, and carotid stenosis degree were found to be risk factors for ischemic vascular events [ 22]. Also, a study using doppler ultrasonography to assess global cerebral inflow discovered that individuals with decreased cerebral blood flow were more likely to develop ischemia, providing a novel and simple way for detecting patients at risk of cerebral ischemia [23]. However, ultrasound is used primarily in assessing the echogenicity of plaques and has lower sensitivity for detecting plaque ulceration when compared to CT and MR angiography [6]. Future we could enhance our database with more CT and MR angiography.

Our study has the following limitations. First, this study used a single-center database and had a small sample size, which may increase the possibility of selection bias. Second, transesophageal echocardiography and a long-term electrocardiogram were not conducted, which may slightly overestimate the effect of non-stenotic carotid plaque on ESUS.

## Conclusion

The clinical characteristics of ESUS were more similar to those of LAA than of CE. Both non-stenotic carotid plaque and vulnerable non-stenotic carotid plaque were more prevalent on the ipsilateral side of ESUS than CE. The presence of ipsilateral vulnerable non-stenotic carotid plaque played an important role in diagnosing ESUS. Our findings support the view that non-stenotic carotid plaque is significantly associated with ESUS. Carotid ultrasonography should be used more broadly to screen for carotid plaque in the ESUS population.

## Data Availability

The datasets collected and/or analyzed during the current study are available upon reasonable request from the corresponding author.

## Abbreviations

ESUS: Embolic stroke of undetermined source
CE: Cardiogenic embolism
LAA: Large-artery atherosclerosis
OR: Odds ratio
CI: Confidence intervals
SD: Standard deviation
IQR: Interquartile range
TOAST: Trial of org 10,172 in acute stroke treatment
CT: Computed tomography
MRI: Magnetic resonance imaging
ECG: Electrocardiogram
TG: Triglyceride
TCH: Total cholesterol
LDL-C: Low density lipoprotein
NIHSS: National Institutes of Health Stroke Scale
ROC: Receiver-operating characteristic
